# Electrochemical Stability of Rhodium–Platinum Core–Shell Nanoparticles: An Identical Location Scanning Transmission Electron Microscopy Study

**DOI:** 10.1021/acsnano.3c04039

**Published:** 2023-08-21

**Authors:** Miquel Vega-Paredes, Raquel Aymerich-Armengol, Daniel Arenas Esteban, Sara Martí-Sánchez, Sara Bals, Christina Scheu, Alba Garzón Manjón

**Affiliations:** † Max-Planck-Institut für Eisenforschung GmbH (MPIE), Max-Planck-Straße 1, 40237 Düsseldorf, Germany; ‡ Electron Microscopy for Materials Science (EMAT), University of Antwerp, 2020 Antwerp, Belgium; § Catalan Institute of Nanoscience and Nanotechnology (ICN2), CSIC and BIST, Campus UAB, 08193 Bellaterra, Spain

**Keywords:** fuel cells, catalysts, identical location, degradation, platinum, rhodium, core−shell

## Abstract

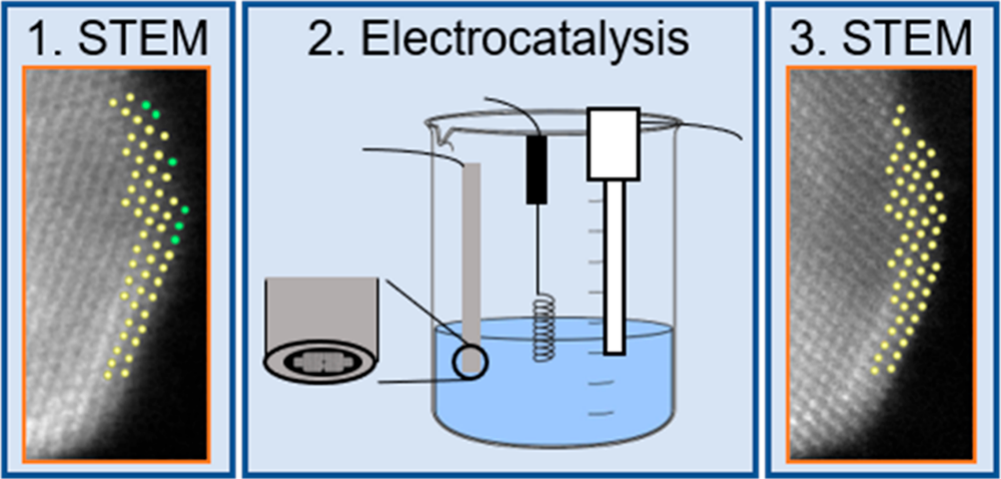

Rhodium–platinum core–shell nanoparticles on a carbon support (Rh@Pt/C NPs) are promising candidates as anode catalysts for polymer electrolyte membrane fuel cells. However, their electrochemical stability needs to be further explored for successful application in commercial fuel cells. Here we employ identical location scanning transmission electron microscopy to track the morphological and compositional changes of Rh@Pt/C NPs during potential cycling (10 000 cycles, 0.06–0.8 V_RHE_, 0.5 H_2_SO_4_) down to the atomic level, which are then used for understanding the current evolution occurring during the potential cycles. Our results reveal a high stability of the Rh@Pt/C system and point toward particle detachment from the carbon support as the main degradation mechanism.

Pt–Rh-based materials have attracted a great deal of attention in the last years due to their wide range of catalytic applications, including the preferential oxidation of CO in hydrogen,^1,2^ the control of NO_*x*_ and CO emissions from car exhaust,^3^ the hydrogen evolution reaction,^4^ formic acid oxidation,^5,6^ and the hydrogenation of organic compounds,^7,8^ among others. Furthermore, Pt–Rh-based materials have been explored as catalysts for proton exchange membrane fuel cells (PEMFCs), both in the cathode^9^ and in the anode, where they have been investigated for the oxidation of methanol^10,11^ and ethanol^10−12^ or as CO-tolerant hydrogen oxidation reaction (HOR) catalysts.^13,14^

Despite these investigations, the understanding of the electrochemical stability and degradation mechanisms affecting Pt–Rh catalysts and the resulting impact on their catalytic activity remains limited. This is especially relevant for PEMFCs, since limited catalyst stability is one of the central factors limiting their widespread commercialization.^15^ Particle dissolution, agglomeration, Ostwald ripening, and particle detachment have been reported as some of the main phenomena affecting state-of-the-art Pt-based PEMFC catalysts.^16^ Moreover, when Pt is alloyed with a less stable transition metal, such as Ni or Co, the preferential dissolution of these metals can take place.^17,18^ This process also affects Pt–Ru-based catalysts,^19−21^ the materials of choice for CO-tolerant PEMFC anodes due to their superior catalytic activity,^22,23^ which impacts greatly their performance toward the HOR. Therefore, it is still necessary to find a durable anode catalyst that can boost the commercial viability of PEMFCs.^22^ In this context, Pt–Rh catalysts are promising candidates to replace Pt–Ru-based anodes on PEMFCs if their stability under operating conditions is higher, as hinted by the Pourbaix diagrams of Ru and Rh.^24^ However, their electrochemical stability needs to be further explored since not much is known about the degradation mechanisms affecting Pt–Rh catalysts under fuel cell conditions.

In this work we study the stability of Rh–Pt core–shell nanoparticles on a turbostratic carbon support (Rh@Pt/C NPs) during electrochemical cycling (10 000 cycles, 0.06–0.8 V_RHE_, 0.5 H_2_SO_4_) by identical location scanning transmission electron microscopy (IL-STEM).^25^ This particular nanostructure was selected because similar core–shell NPs have been shown to have a superior performance than the alloyed counterparts.^1,26,27^ IL-(S)TEM is a powerful tool for studying local changes down to the atomic level in nanostructured catalysts, which has been extensively used for gaining insights into the degradation of electrocatalysts^28−32^ or their support^29,33^ under fuel cell conditions. In IL-(S)TEM, the same region of interest can be investigated before and after electrochemical testing, allowing correlation of the changes in the particles with the catalytic activity. This technique solves some of the limitations of ex situ (S)TEM, in which only general statistical insights are possible, which might fail to reflect the exact changes taking place in the system.^34^ In addition, it also presents some advantages compared with in situ electrochemical liquid cell (S)TEM, in which the electron beam induced radiolysis of the electrolyte can produce unwanted artifactual reactions,^35^ as well as having its special resolution limited by the presence of a thick liquid layer.^36^

Our results indicate that the investigated Rh@Pt/C NPs are stable systems and point toward particle detachment as the main degradation mechanism taking place during potential cycling.

## Results and Discussion

### Characterization of the As-Synthesized Rh@Pt NPs

**Figure 1:**
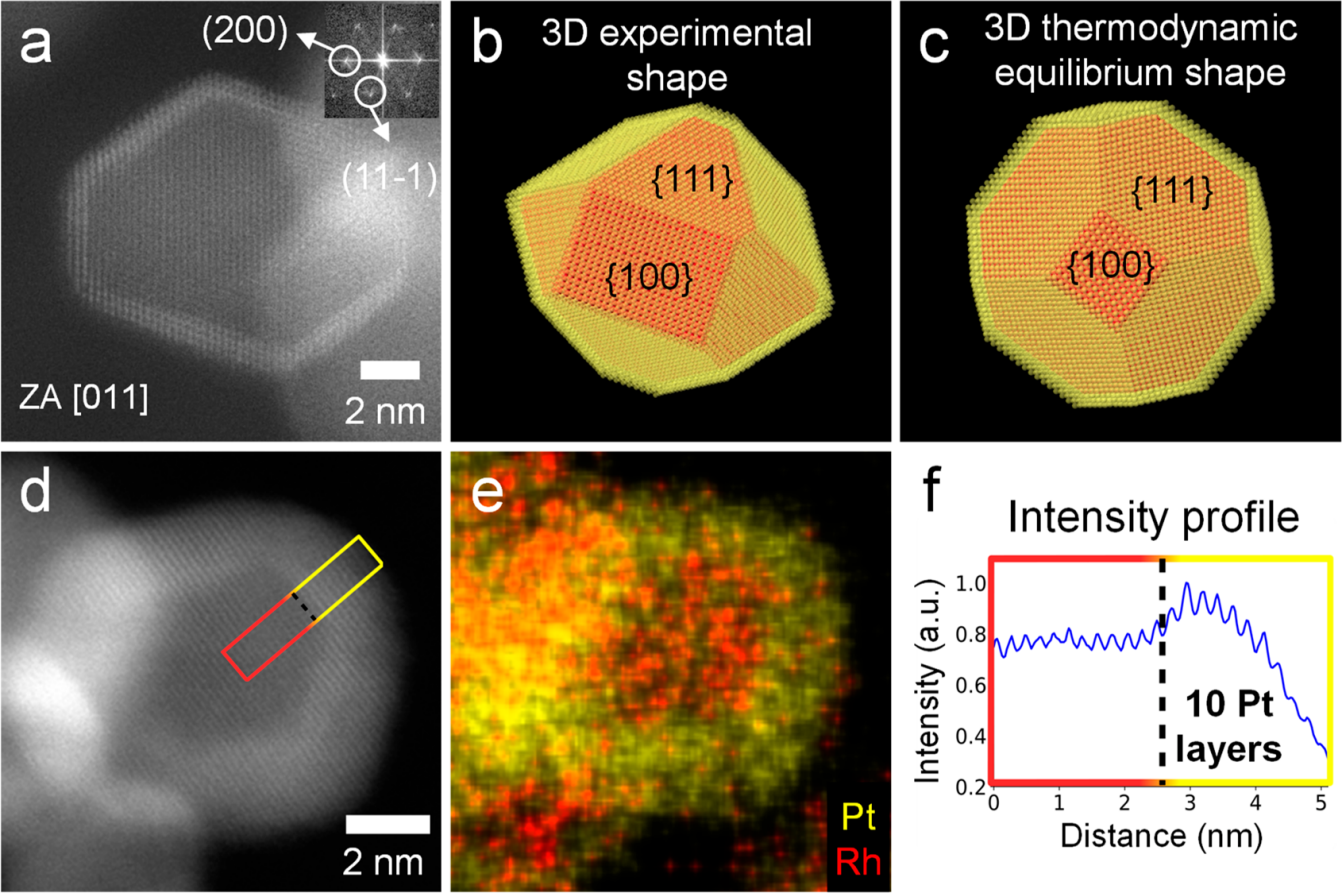
Structural and compositional characterization of the as-synthesized Rh@Pt/C NPs. (a) HAADF-STEM micrograph of a faceted particle visualized along the [011] ZA. Its FFT is shown as an inset. (b) 3D atomic model of that particle that deviates from the thermodynamic equilibrium shape, shown in (c). (d) HAADF-STEM micrograph, (e) its corresponding energy dispersive X-ray spectroscopy (EDS) composition map, and (f) intensity profile along a NP with a 10-monolayer-thick Pt shell.

The as-synthesized Rh@Pt/C NPs were characterized by means of (S)TEM in order to confirm the core–shell structure and study the atomic arrangement (Figure 1). The higher atomic number of Pt compared to Rh (Z_Pt_ = 78, Z_Rh_ = 45) results in Pt atoms scattering electrons more strongly and appearing brighter in the high angle annular dark field (HAADF) images.^37^ Therefore, the bright shell surrounding the dark core in the particle in Figure 1a is indicative of a Rh-core Pt-shell particle. The fast Fourier transform (FFT; inset Figure 1a) shows that both the shell and the core are face-centered cubic and oriented along the [011] zone axis (ZA). The Rh@Pt/C NPs investigated in this work are highly faceted and present the lowest energy {111} and {100} facets (Supporting Information (SI) Figure FS1).^38^ However, their shape deviates from that of the thermodynamic equilibrium (Figure 1b,c), as they present asymmetric facets that distort the 3D structure from the expected cuboctahedron. The shell thickness is measured to be between three (Figure 1a) and 10 (Figure 1d–f) Pt monolayers. Nonetheless, even in the particles with a shell of only three Pt monolayers, the Rh core appears to be fully encapsulated, which is desirable since an incomplete core coverage would result in the less stable Rh being exposed to the electrolyte during cycling/operation, and therefore in a lower stability.^20^

**Figure 2:**
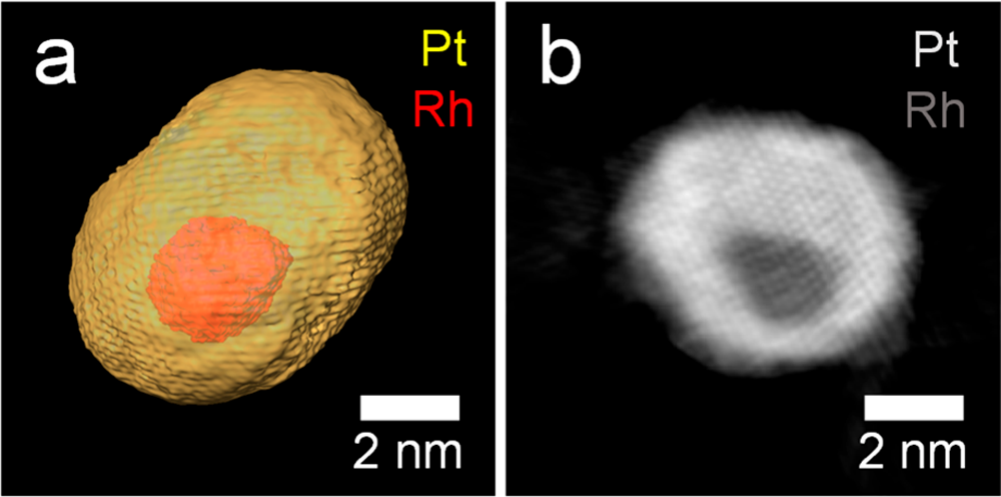
High-resolution tomography of as-synthesized Rh@Pt/C NPs. (a) Segmented 3D volume and (b) orthoslice of the reconstruction.

Since the encapsulation of the core is a 3D phenomenon, the NPs were further investigated by high-resolution electron tomography (Figure 2). In Figure 2a, the intensity-based segmentation of a reconstructed Rh@Pt/C NP at atomic resolution is shown. The low-intensity voxels are assigned to Rh, whereas those with high intensity correspond to Pt (Figure 2b). An animated version of the reconstruction and orthoslices through the 3D data set can be found in the SI. These results confirm that the Rh core is completely surrounded by the Pt shell, and therefore a high electrochemical stability can be expected.

### Electrochemical Accelerated Stress Tests

After the initial characterization of the Rh@Pt NPs, accelerated stress tests (ASTs) were carried out, as described in the Experimental Methods. The upper potential limit of 0.8 V_RHE_ differs from ASTs found in the literature, in which higher upper potential limits are used for studying the degradation associated with start-up or shut-down events.^30,39^ However, the development of system strategies mitigates the degradation during these events,^40,41^ making a lower upper potential limit more suitable for studying anode catalyst degradation studies.^21^

**Figure 3:**
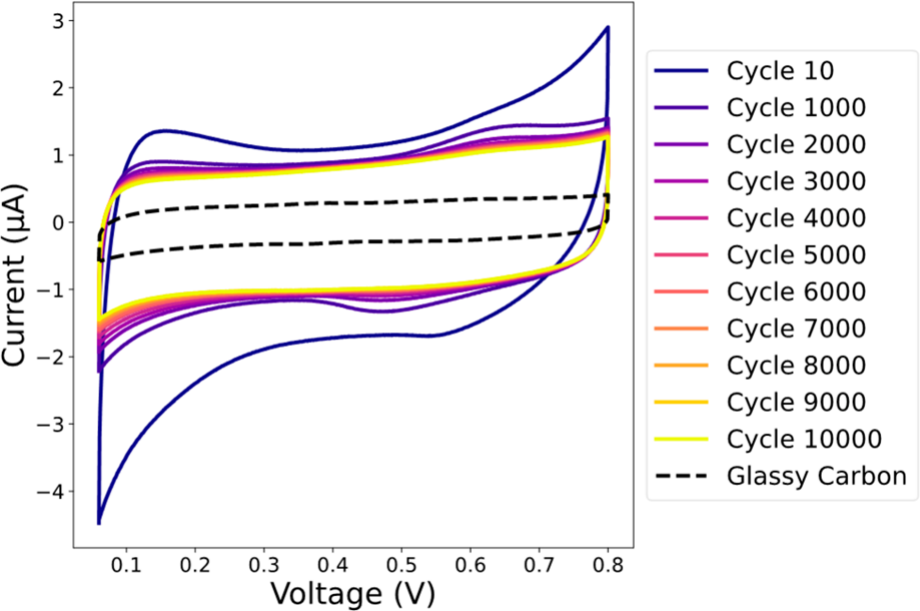
Voltammograms showing the changes in the Rh@Pt/C NPs’ activity during the potential cycles.

Figure 3 shows the changes in the Rh@Pt/C-TEM grid voltammograms that occurred during the potential cycles. A cyclic voltammetry recorded with the bare glassy carbon electrode is also provided as blank current. The comparatively higher current obtained after adding the TEM grid confirms the correct electrical connection of the grid to the electrode.

In the initial cycles oxidation and reduction peaks are present at 0.65 V_RHE_ and 0.55 V_RHE_, respectively, which can be attributed to Pt.^42^ Additionally, a peak in the hydrogen underpotential deposition region is also seen. During the potential cycles, the cyclic voltammetry curves flatten progressively, and after 10 000 cycles only capacitive current can be observed, which can be attributed to the carbon support.^43^ This is indicative of a loss of active catalyst during the potential cycles, which could be caused by the common degradation mechanisms of fuel cell catalysts (particle dissolution, agglomeration, Ostwald ripening, and particle detachment from the carbon support). Moreover, since the cycles are performed on a TEM grid loaded with Rh@Pt/C NPs without any binder, large groups of Rh@Pt/C NPs not properly attached to the TEM grid can also get removed (Figure FS2), which partially explains the loss of current observed in Figure 3. Nonetheless, loss of current is still observed for the voltammograms performed on Rh@Pt/C-glassy carbon (Figure FS3a) to a smaller extent. Since that electrode contained Nafion as a binder, the removal of large groups of Rh@Pt/C NPs cannot solely explain the loss of activity of Rh@Pt/C NPs, and other degradation mechanisms need to be considered.

### Identical Location STEM

**Figure 4:**
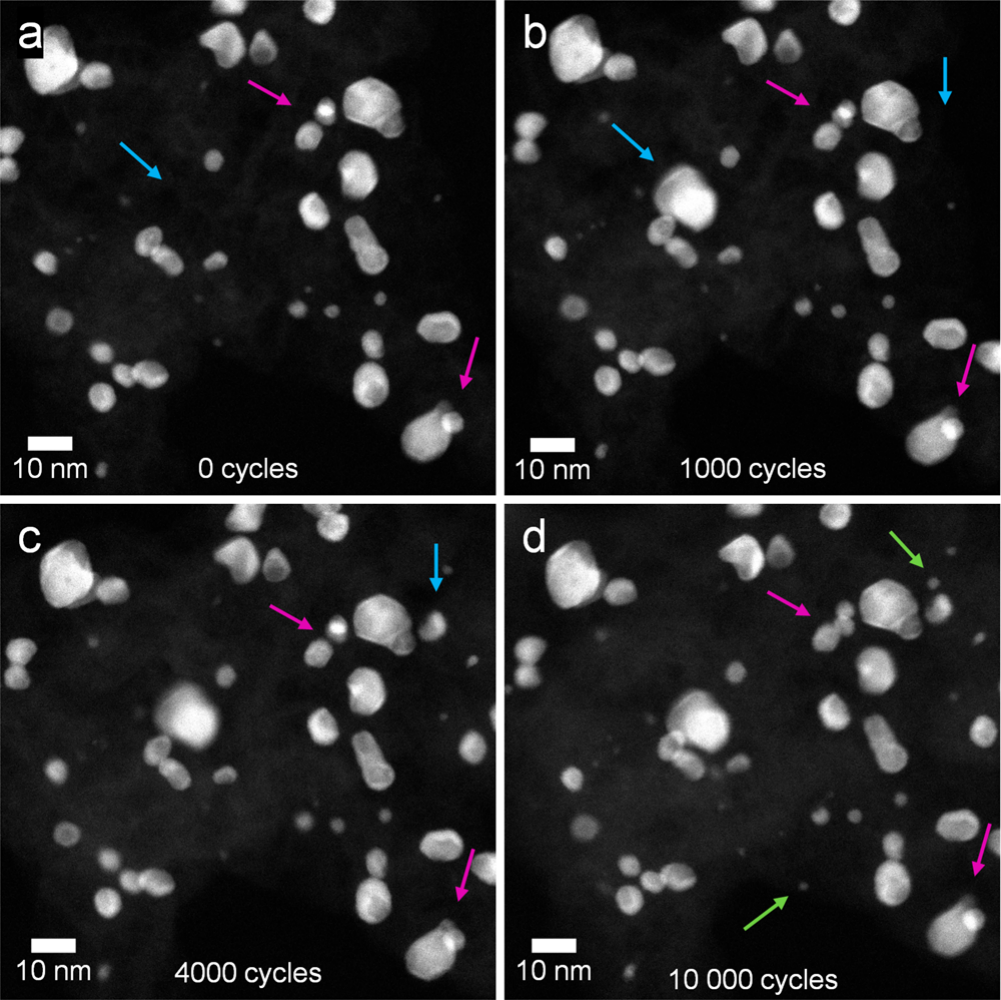
HAADF-STEM micrographs showing a region of the sample at (a) 0, (b) 1000, (c) 4000, and (d) 10 000 AST cycles. The different colored arrows indicate particle movement (purple), particle reattachment from another region (blue), and particle reprecipitation from dissolved species (green).

In order to understand the causes behind the current degradation observed in Figure 3, IL-STEM experiments were performed. Figure 4 shows how a representative region of the sample changed during the potential cycles. In the SI, the evolutions of other regions are provided (Figures FS4, FS5). No clear particle growth or agglomeration can be seen, and a constant mean particle size (represented by the equivalent diameter) of ∼5.9 nm is found during the cycles. The particle size distribution histograms are provided in the SI (Figure FS6). A similar particle size distribution was found for the particles cycled directly after drop casting on the glassy carbon electrode (Figure FS3d). The fact that the mean particle size does not increase during ASTs is indicative of the stability of the particles under cycling conditions. Thus, the observed loss of current of the catalytic material cannot be attributed to a decrease in the electrochemically active surface area (ECSA) of the catalysts derived from NP growth. Nonetheless, small-particle nucleation is observed after 10 000 cycles (Figure 4, green arrows). Although it is possible that part of the small particles could be originating from redeposition of Pt ionic species dissolved from the Pt-wire counter electrode, small-particle nucleation was also observed when a glassy carbon counter electrode was used (Figure FS7). The presence of small Pt particles was also reported in previous IL-STEM studies on Pt–Ni NPs and was attributed to the dissolution and reprecipitation of catalytic species.^44^ Although the authors claimed that these dissolved species could migrate on the carbon support and redeposit onto other particles, resulting in Ostwald ripening, this is not observed in the mean particle size of the Rh@Pt/C NPs, which indicates that this Ostwald ripening is not significant in our system for the chosen conditions. Besides the constant mean particle size, X-ray diffraction experiments on the Rh@Pt/C NPs (Figure FS3b) also reveal that the crystalline structure of the NPs did not change during the potential cycles.

During all of the AST cycles, particle movement (Figures 4, FS4, and FS5, purple arrows) can be observed. This is a well-documented phenomenon occurring in PEMFCs,^45−48^ which is caused by changes in the underlaying carbon support (i.e., the turbostratic carbon). Carbon corrosion is thermodynamically possible at voltages *E* > 0.207 V_RHE_, and although it is kinetically slow at typical PEMFC potentials, it is known to be catalyzed by the Pt present in the catalyst NPs.^48^ This particle movement can result in particle aggregation and coalescence, which decrease the ECSA of the catalyst and can be one of the main degradation mechanisms behind the loss of performance in PEMFCs. However, the particle movement observed for the Rh@Pt/C NPs during the potential cycles is subtle. Since the movement can take place both in and out of plane, from the 2D projections, it cannot be properly quantified. To assess if the Rh@Pt/C NPs have a tendency of decreasing their distance and would eventually get aggregated if more cycles are performed, low-magnification HAADF-STEM tomography experiments at different points of the potential cycles (0, 1000, 4000, 10 000) were carried out. In the SI, the animated movies of the segmented reconstructed volumes are provided. Similarly to the HAADF micrographs, the low-magnification 3D reconstructions also show particle movement during the potential cycles, which results in small fluctuations in the average nearest neighbor distance (Figure FS8). However, no clear trend can be discerned, meaning that the Rh@Pt/C NPs are not significantly aggregated during the ASTs. Therefore, particle aggregation/agglomeration can be excluded as the main degradation mechanisms of these particles.

**Figure 5:**
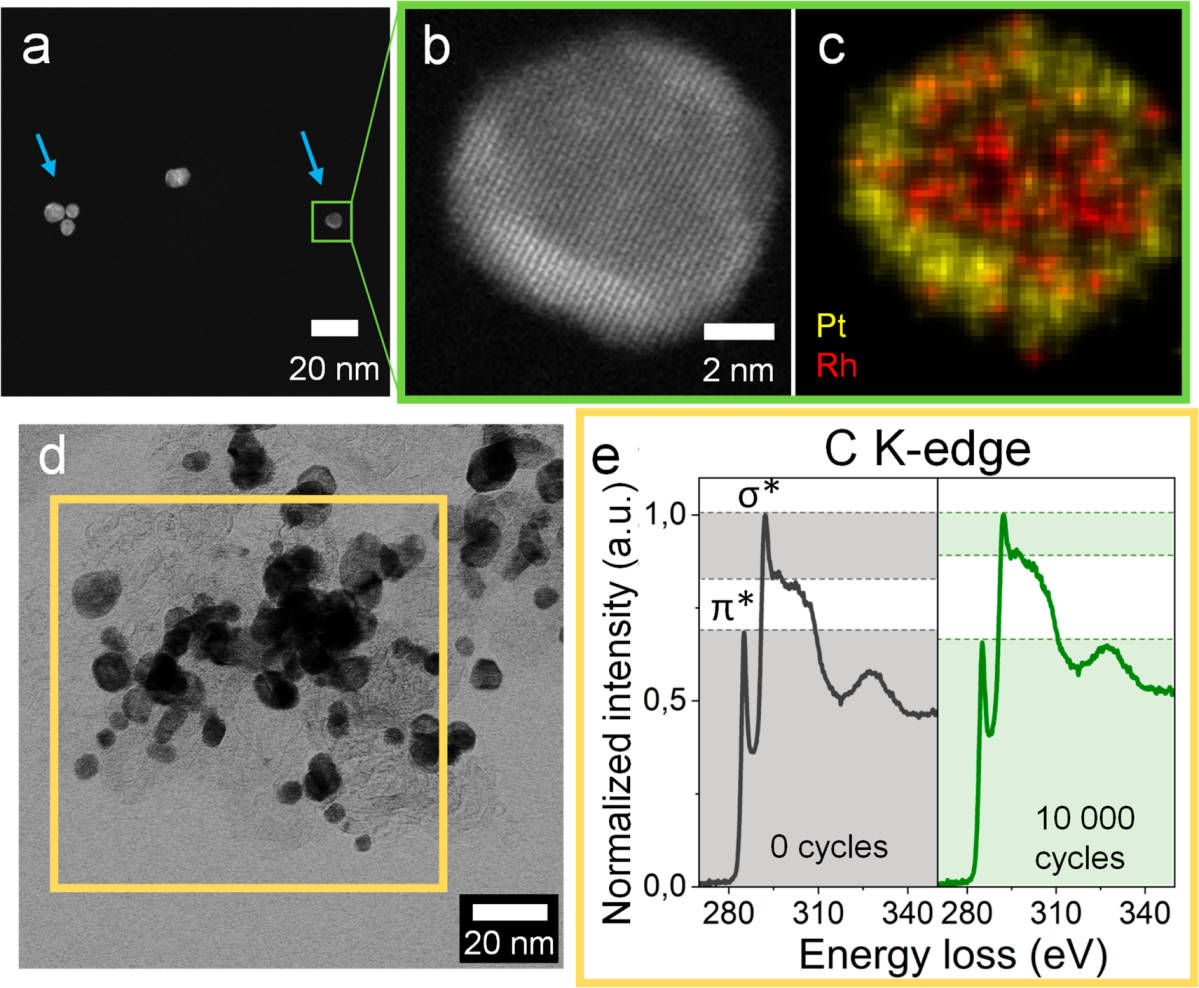
Particle detachment due to C support corrosion. (a) Rh@Pt particles found after 1000 ASTs without a carbon support (blue arrows) that have reattached from other regions of the sample. HAADF micrograph (b) and EDS composition map (c) of one particle. (d) Bright-field STEM micrograph, showing the region where the EELS spectra (e) were taken at 0 and 10 000 cycles.

Besides particle movement, another consequence of the turbostratic carbon support corrosion is particle detachment, which results in the loss of catalyst material with the corresponding drop in ECSA and PEMFC performance. During IL-STEM experiments, the detached particles can (i) reattach in another region containing Rh@Pt/NPs, (ii) reattach on the TEM grid, and (iii) be washed out by the electrolyte. Examples of particle reattachment from a different region can be seen in Figure 4 (blue arrows), where particles that are not present at 0 cycles appear after 1000 or 4000 cycles. Lower magnification images were also checked to discard the possibility of particle migration from a neighboring region. Particle reattachment on the TEM grid can also be observed frequently, since after potential cycling Rh@Pt particles without a turbostratic carbon support can be found on the TEM grid (Figure 5a–c). Considering that all of the particles were found deposited on the carbon support before the cycles, this is a clear indication of particle detachment from the carbon support and reattachment on the TEM grid. To see better the lack of turbostratic carbon support, intensity saturated micrographs of the area depicted in Figure 5 are provided in the SI (Figure FS9), together with carbon-supported Rh@Pt/C NPs for comparison. Notice that when the carbon support is present, it can be clearly distinguished from the amorphous carbon from the TEM due to the presence of graphitic (0001) planes.

In the SI (Figure FS10), two examples of particles being detached from the turbostratic carbon support are provided. These results indicate that particle detachment from the carbon support is a relatively frequent phenomenon in Rh@Pt/C NPs. The degradation of the carbon support was further confirmed by electron energy loss spectroscopy (EELS; Figure 5d,e). The EELS spectrum of the carbon K-edge before cycling is typical for graphitic carbon materials, with peaks corresponding to transitions to antibonding π* states (∼285 eV) and antibonding σ* states (∼292 eV).^49^ After 10 000 cycles, the intensity of these peaks decreased, demonstrating an amorphization of the carbon support.^50^ Nonetheless, pronounced π* and σ* peaks are still observed, indicating that the amorphization is partial and explaining the moderate particle movement and detachment observed in the IL-STEM experiments. A similar partial amorphization was seen in a region not previously exposed to the electron beam (Figure FS11), ruling out possible electron beam effects, and for the carbon support on the Rh@Pt/C NPs cycled directly after drop casting on the glassy carbon electrode (Figure FS3e).

As previously discussed, no particle aggregation/agglomeration, significant catalyst dissolution, or Ostwald ripening (Figure 4, Figure FS6) is observed in the IL-STEM experiments. Therefore, the lower current observed in Figure 3 and Figure FS3a upon electrochemical cycles is assigned to be predominantly caused by particle detachment from the turbostratic carbon support. Efforts for increasing the stability of Rh@Pt/C particles should focus on enhancing the support–NP interaction, which could be achieved by functionalizing the support^51^ or by replacing it with another material, such as oxide-^52^ or graphene^53^-based supports.

**Figure 6:**
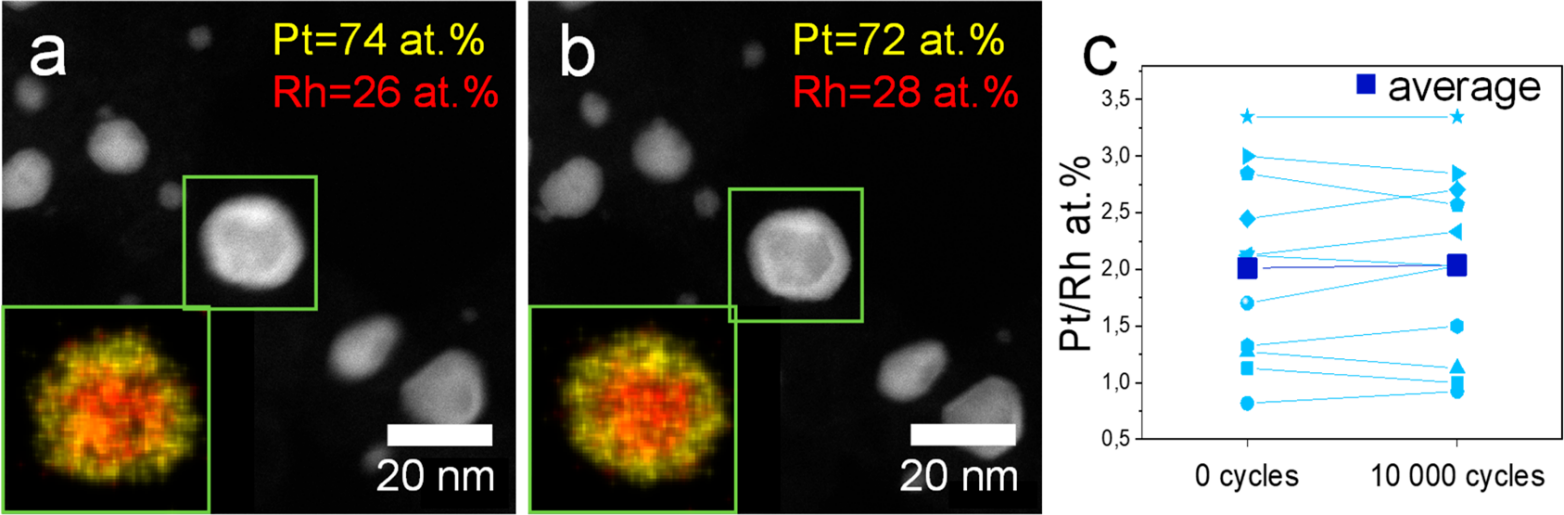
Composition changes in Rh@Pt/C NPs during ASTs. HAADF-STEM micrographs of the same region are shown at 0 (a) and 10 000 (b) potential cycles. As insets, the EDS composition maps of the central NP are provided. (c) Evolution of the Pt/Rh at. % ratio quantified from the EDS composition maps for 11 different particles.

Besides the previously mentioned degradation mechanisms, in bimetallic systems, other phenomena can occur. For instance, in bimetallic Pt–Ru particles preferential dissolution of Ru can take place.^19^ Even in core–shell NPs, Ru-core dissolution can take place if the Pt-shell is not fully covering the core^20^ or by thermally induced shape fluctuations of the shell,^54^ both of which result in the less stable core metal being exposed to the electrolyte, with its consequent dissolution. Energy dispersive X-ray spectroscopy (EDS) spectral images were acquired during the ASTs to study the changes in the elemental composition and distribution of the Rh@Pt/C NPs. In Figure 6, the elemental distribution maps of a Rh@Pt/C NP are shown. EDS maps of other particles are also provided in the SI (Figure FS12). These results reveal an identical core–shell structure before and after the ASTs, indicating the compositional stability of the NP system. Moreover, no Rh preferential dissolution is detected, since the composition of the particles remains unaltered during potential cycling, with only small variations of up to 3 at. % (near the sensitivity of the technique) that do not follow a clear trend (Figure 6c). This is in good agreement with the HR-STEM observations and EDS composition maps performed on particles cycled directly after drop casting on the glassy carbon electrode (Figure FS3c), which further confirm the preservation of the core–shell structure.

This was further confirmed in lower magnification EDS experiments in order to increase the statistics in the measures (Figure FS13). Figure FS13 corroborates that there are no significant changes in the elemental composition during the ASTs. This is most likely caused by the full Rh core encapsulation (Figure 1, Figure 2) and by the higher electrochemical stability of Rh than Ru.^24^

**Figure 7:**
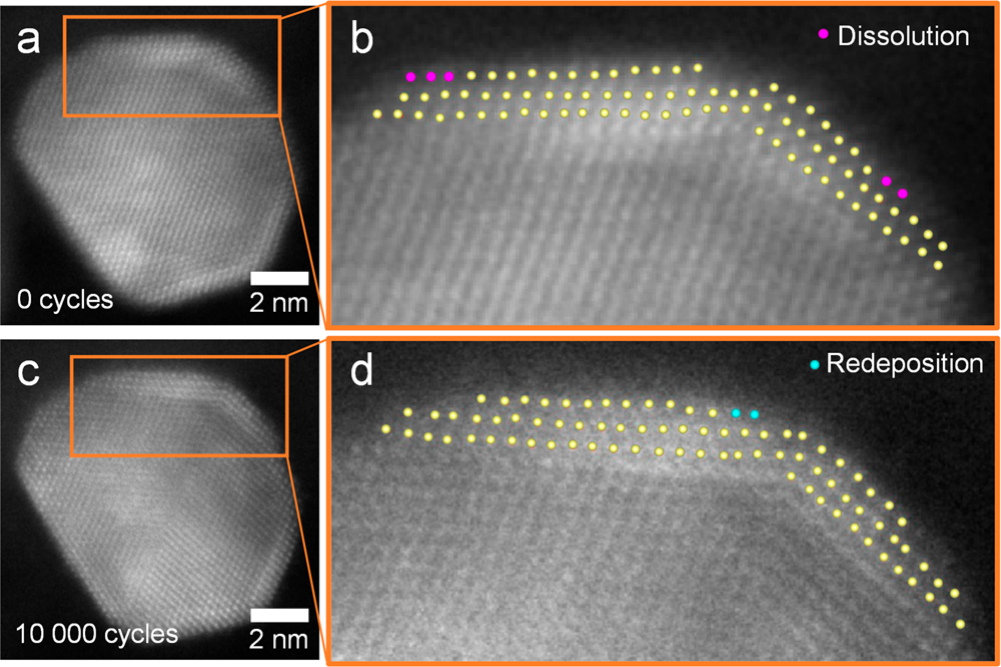
Atomic scale changes in Rh@Pt/C NPs after 10 000 cycles. The same particle at 0 (a, b) and 10 000 (c, d) cycles is shown. The atomic column positions of the outermost three atomic layers are indicated by yellow dots. The pink dots indicate those atomic columns that dissolved during the ASTs, while the blue dots indicate those that got redeposited.

Even though the size and composition of Rh@Pt/C NPs remained practically constant during potential cycles, a careful analysis of high-resolution STEM micrographs shows that changes at the atomic level took place (Figure 7, Figure FS14). Both dissolution (i.e., atoms present at 0 cycles that are not present after 10 000 cycles) and redeposition (i.e., atoms not present in the as-synthesized particle, that appear after the ASTs) of atomic columns can be observed. These two phenomena occur in the surface steps involving atoms with unsaturated bonds. A similar behavior was reported by Rasouli et al.^44^ for Pt–Ni NPs in more oxidative conditions (0.6–1.1 V_RHE_), in which the atoms on high-energy sites (steps and kinks) dissolved during potential cycling. Our results indicate that in the case of Rh@Pt/C NPs atomic column dissolution can take place, even under the milder conditions used for our ASTs. Beyond dissolving in the electrolyte, these species can either redeposit on previously existing particles or nucleate forming small particles, as seen in Figure 4 (green arrows), explaining their origin.

To sum up, our results indicate that Rh@Pt/C NPs are very stable systems under the studied conditions, as opposed to comparable Ru@Pt/C NPs.^20,21^ Moreover, they also point out that particle detachment from the turbostratic carbon support is the main phenomenon causing the loss of active catalyst material in Rh@Pt/C NPs during ASTs. Therefore, efforts for increasing the stability should focus on enhancing the carbon support–particle interaction or on different support materials.

## Conclusions

In the present work, the stability of Rh@Pt/C NPs obtained via a two-step polyol synthesis was explored. After initial characterization of the particles showing a complete Rh-core encapsulation, accelerated stress tests in the form of potential cycles (0.06–0.8 V_RHE_, 10 000 cycles) were performed on a bulk electrode and on a TEM grid. The loss of activity seen in the voltammograms during the potential cycles was correlated to the morphological and compositional changes observed on the TEM grid in IL-STEM experiments.

The main degradation mechanism affecting the Rh@Pt/C NPs was found to be particle detachment from the carbon support. Although atomic column dissolution and redeposition were observed, no significant changes in the particle size or composition were detected.

Our results indicate that Rh@Pt/C particles are very stable under the tested electrochemical conditions and point that efforts for improving even further the stability should focus toward enhancing the support–particle interaction.

## Experimental Methods

### Rh@Pt/C NP Synthesis

Carbon-supported Rh@Pt/C NPs were prepared via a two-step polyol synthesis. The detailed synthesis protocol has been previously described elsewhere.^21^ A nominal catalyst loading of 21.1 wt % Rh and 20.0 wt % Pt was used. The carbon support was Cabot FCX 400.

### Electrochemical Cycling

To study the degradation of the Rh@Pt/C NPs, these were subjected to ASTs by cyclic voltammetry acquired in a three-electrode setup. As counter and reference electrodes, a Pt wire (Redoxme) and a reversible hydrogen electrode (RHE, Gaskatel) were respectively used. The system was controlled by a Gamry 600 reference potentiostat. Before the ASTs, a 0.5 M sulfuric acid electrolyte (H_2_SO_4_, Suprapur, Sigma-Aldrich) was purged for 30 min with argon. The potential window was chosen between 0.06 V_RHE_ and 0.8 V_RHE_, and a total of 10 000 cycles were carried out, conditions previously used for studying the degradation of an anode catalyst in PEMFCs.^20,21^

The Rh@Pt/C-glassy carbon working electrode was obtained by drop casting 10 μL of an ink (1 mg of Rh@Pt/C + 30 μL of Nafion + 2 mL of isopropanol) in a polished glassy carbon electrode. After the cycles, the electrode was scratched to detach some of the cycled Rh@Pt/C NPs, which were later characterized using X-ray diffraction (XRD) and STEM.

Additionally, to study how individual Rh@Pt/C NPs changed during potential cycles in identical location conditions, 10 μL of a 0.28 mg/mL dispersion of Rh@Pt/C on deionized (DI)-water (0.055 μS/cm) was drop cast onto a holey carbon-coated Au TEM finder grid and left drying overnight. After initial characterization of the Rh@Pt/C NPs, this TEM grid was fixed on a glassy carbon electrode with a holey Teflon cap and used as a working electrode on the same three-electrode setup. This allowed for tracking changes in specific regions (and particles within those regions) between 0, 1000, 4000, and 10 000 potential cycles.

### (S)TEM Characterization

High-resolution (HR)-TEM micrographs of the as-synthesized Rh@Pt/C NPs were obtained in an image-corrected Titan Themis microscope (Thermo Fisher Scientific) operated at 300 kV. The corrector was set to introduce negative spherical aberration on the objective lens, which together with an overfocus results in bright atom contrast.^55^ The TEM images were recorded on a CMOS 4k × 4k camera.

The Rh@Pt/C NPs were characterized as synthesized and after 1000, 4000, and 10 000 potential cycles by means of STEM. STEM micrographs were acquired in a probe-corrected Titan Themis microscope (Thermo Fisher Scientific) operated at 300 kV by using an HAADF detector. A convergence angle of 23.8 mrad was used, resulting in a probe of around 0.1 nm. 3D atomic models were constructed from the STEM micrographs using the Rhodius software.^56,57^ The equilibrium thermodynamic shape was built taking as a reference the Wulff construction for Rh.^58^ EDS spectral images were acquired to study the chemical composition and elemental distribution of Rh@Pt/C NPs. To minimize the electron-beam-induced damage on the particles, the acquisition time of the EDS spectral images was limited to 5 min and were only taken on the as-synthesized NPs and at the end of the AST (after 10 000 cycles). Moreover, HAADF micrographs before and after the acquisition were taken and compared to rule out electron-beam-induced morphological changes. Quantification from the EDS spectral images was performed with the Cliff-Lorimer method.^59^ EELS data were acquired in STEM mode with a dispersion of 0.100 eV per channel and a pixel acquisition time of 1 s using the Quantum Gatan imaging filter. All of the spectra were acquired on NPs lying on holes of the TEM grid, to avoid the contribution from the carbon of the grid. For the high-resolution STEM-HAADF tomography, a “cubed” aberration-corrected Thermo Fisher Titan X-Ant-EM operating at 300 kV was utilized to acquire the HAADF-STEM projections. In addition, to study the nearest neighbor distance variation during potential cycles, low-magnification tomography was performed. More experimental details can be found in the Supporting Information.

### X-ray Diffraction Experiments

The XRD experiments were performed on a Rikaku Smartlab 9kw diffractometer, using a micro area optics setup and Cu Kα radiation as X-ray source. The cycled particles for the XRD experiments were obtained by scratching the cycled Rh@Pt/C-glassy carbon working electrode.

## Supporting Information Available

Additional details on tomography experiments and supplementary figures referenced in the manuscript (DOCX)
Animated movie with atomic 3D model of experimental particle (AVI)
Animated movie with atomic 3D model of particle in thermodynamic equilibrium (AVI)
Animated movie with segmented high-resolution tomographic reconstruction and orthoslice cut of as-synthesized Rh@Pt/C NPs (MP4)
Animated movie with segmented low-resolution tomographic reconstruction of Rh@Pt/C NPs at 0 potential cycles (AVI)
Animated movie with segmented low-resolution tomographic reconstruction of Rh@Pt/C NPs at 1000 potential cycles (AVI)
Animated movie with segmented low-resolution tomographic reconstruction of Rh@Pt/C NPs at 4000 potential cycles (AVI)
Animated movie with segmented low-resolution tomographic reconstruction of Rh@Pt/C NPs at 10 000 potential cycles (AVI)

## Supplementary Material

nn3c04039_si_001.docx

nn3c04039_si_002.avi

nn3c04039_si_003.avi

nn3c04039_si_004.mp4

nn3c04039_si_005.avi

nn3c04039_si_006.avi

nn3c04039_si_007.avi

nn3c04039_si_008.avi
