# Supplementary material for: Electrochemical Stability of Rhodium–Platinum Core–Shell Nanoparticles: An Identical Location Scanning Transmission Electron Microscopy Study
Source: ACS Nano. 2023 Aug 21;17(17):16943–51. doi: 10.1021/acsnano.3c04039 (PMC10510721; doi:10.1021/acsnano.3c04039)
Supplement: Supplementary file 1 — nn3c04039_si_001.docx [file nn3c04039_si_001.docx]

Supplementary Information for:

Electrochemical Stability of Rhodium-Platinum Core-Shell Nanoparticles: An Identical Location Scanning Transmission Electron Microscopy Study

Miquel Vega-Paredes^1*^, Raquel Aymerich-Armengol^1^, Daniel Arenas Esteban^2^, Sara Martí-Sánchez^3^, Sara Bals^2^, Christina Scheu^1*^, Alba Garzón Manjón^1,3*^

^1^ Max-Planck-Institut für Eisenforschung GmbH (MPIE), Max-Planck-Straße 1, 40237 Düsseldorf, Germany

^2^ Electron Microscopy for Materials Science (EMAT), University of Antwerp, 2020 Antwerp, Belgium

^3^ Catalan Institute of Nanoscience and Nanotechnology (ICN2), CSIC and BIST, Campus UAB, 08193 Bellaterra, Spain

*Corresponding authors:

[m.vega@mpie.de](mailto:m.vega@mpie.de)

[c.scheu@mpie.de](mailto:c.scheu@mpie.de)

[alba.garzon@icn2.cat](mailto:alba.garzon@icn2.cat)

**Experimental details on the STEM-HAADF tomography experiments**

The acquisition of the high-resolution tomography of the as synthesized NPs was performed following a specific methodology to minimize the beam damage. Projections were acquired using a Fischione model 2020 single tilt holder over a tilt range of ±72° with angular increments of 3°. At each tilt, 5 frames were acquired with a 1.5 µs dwell time, while the image resolution was set to 2048x2048 pixels. Next, we used these images as input for a non-rigid registration method in combination with a convolutional neural network (CNN) ^1^. The obtained series were aligned using cross-correlation and 3D reconstructions were obtained using the Simultaneous Iterative Reconstruction Technique (SIRT) algorithm, as implemented in Astra Toolbox ^2^. This volume was then segmented based on the voxel intensity, to differentiate between the Rh core and the Pt shell. On the other hand, to study the changes of the Rh@Pt/C NPs in 3D at lower magnification, tilt-series between -65° and +65° with angular increments of 5° were acquired with a single tilt holder (Fischione Instruments, Model 2020) using the FEI Tomography software, aligned ^3,4^ and reconstructed using open source Python libraries. The reconstruction was performed using 100 iterations of SIRT, as implemented in TomoPy ^5,6^. The reconstructed volumes were then segmented using the watershed algorithm ^4^ and visualized with Napari ^7^. The nearest neighbour distance was calculated from the location of the central voxel (3D-pixel) in each of the segmented NPs. The relatively big angular increments of 5° during the tilt-series acquisition of the low magnification tomography were chosen as a compromise between number of projections (resolution of the reconstruction) and electron dose received by the NPs, especially considering that several tilt series of the same region at different potential cycles were acquired.

**Supplementary figures**

FS1. HR-TEM micrographs (a,d) and respective FFTs (b,e) of two Rh@Pt/C NPs oriented along the [011] and [001] ZA. In (c,f) the Wulff constructions of Rh along the corresponding zone axis are provided. Notice how the experimental shape of the NPs deviates from that expected on the thermodynamic equilibrium.

*FS2. Detachment of big groups of Rh@Pt/C NPs during the ASTs from the TEM grid due to the absence of a binder (Nafion).*

*FS3. Ex-situ characterization. (a) Voltammograms performed on a Rh@Pt/C NPs ink deposited on a glassy carbon electrode. (b) XRD diffractograms of the Rh@Pt/C NPs at 0 (in gray) and 10 000 (in green) cycles. The diffractograms of Pt (ICSD-76951) and Rh (ICSD-76952) are also provided for comparison.* *The additional reflection at 31.5° is most likely stemming from CaS impuritutes of the carbon support* ^8^*. HR-STEM image, corresponding EDS composition map (c), lower resolution STEM micrograph with particle size distribution histogram (d) and EELS spectra (e) of the Rh@Pt NPs ink after 10 000 cycles.*

FS4. HAADF-STEM micrographs showing a different region of the sample at (a) 0, (b) 1000, (c) 4000 and (d) 10 000 AST cycles. The purple arrows indicate particle movement and the green arrows particle reprecipitation from dissolved species.

FS5. HAADF-STEM images of a different area of the TEM grid at (a) 0, (b) 1000, (c) 4000 and (d) 10 000 AST cycles. The purple arrows indicate particle movement.

FS6. Particle size distribution histograms at 0 (a), 1000 (b), 4000 (c) and 10 000 (d) potential cycles. The mean diameter (µ) and standard deviation (σ) are provided in each histogram.

FS7. Small particle nucleation also takes place when using a glassy carbon counter electrode

*FS8. Segmented tomograms at 0 (a) and 10 000 (b) cycles. (c) Changes in* *the nearest neighbour distance during the ASTs, indicating that despite the particle movement, there is no tendency towards particle aggregation/agglomeration.*

FS9. Saturated HAADF-STEM micrograph of Rh@Pt/C NPs showing the difference between the turbostratic carbon supported NPs (indicated with white arrows) vs particles without carbon support (blue arrows).

*FS10. Particle detachment observed during the IL-STEM experiments in two different regions (a,b).*

FS11. EELS spectra after 10 000 cycles (red) of a region not previously explored with the electron beam. A similar partial amorphization is seen as in the identical location region (green).

*FS12. Composition and elemental distribution changes in Rh@Pt/C NPs during the ASTs.*

FS13. Composition at 0 (a), 1000 (b), 4000 (c) and 10 000 (d) potential cycles. The Pt and Rh atomic ratio from the region delimited by a purple square is provided.

*FS14. Atomic scale changes during potential cycling. The green dots indicate those atomic columns that dissolved during the cycles.*

(1)	Altantzis, T.; Lobato, I.; De Backer, A.; Béché, A.; Zhang, Y.; Basak, S.; Porcu, M.; Xu, Q.; Sánchez-Iglesias, A.; Liz-Marzán, L. M.; Van Tendeloo, G.; Van Aert, S.; Bals, S. Three-Dimensional Quantification of the Facet Evolution of Pt Nanoparticles in a Variable Gaseous Environment. *Nano Lett.* **2019**, *19* (1), 477–481. https://doi.org/10.1021/acs.nanolett.8b04303.

(2)	van Aarle, W.; Palenstijn, W. J.; De Beenhouwer, J.; Altantzis, T.; Bals, S.; Batenburg, K. J.; Sijbers, J. The ASTRA Toolbox: A Platform for Advanced Algorithm Development in Electron Tomography. *Ultramicroscopy* **2015**, *157* (2015), 35–47. https://doi.org/10.1016/j.ultramic.2015.05.002.

(3)	de la Peña, F.; Prestat, E.; Fauske, V. T.; Burdet, P.; Lähnemann, J.; Jokubauskas, P.; Furnival, T.; Nord, M.; Ostasevicius, T.; MacArthur, K. E.; Johnstone, D. N.; Sarahan, M.; Taillon, J.; Aarholt, T.; pquinn-dls; Migunov, V.; Eljarrat, A.; Caron, J.; Francis, C.; Nemoto, T.; Poon, T.; Mazzucco, S.; actions-user; Tappy, N.; Cautaerts, N.; Somnath, S.; Slater, T.; Walls, M.; Winkler, F.; Ånes, H. W. Hyperspy/Hyperspy: Release v1.7.3. Zenodo October 2022. https://doi.org/10.5281/zenodo.7263263.

(4)	van der Walt, S.; Schönberger, J. L.; Nunez-Iglesias, J.; Boulogne, F.; Warner, J. D.; Yager, N.; Gouillart, E.; Yu, T.; the scikit-image contributors. Scikit-Image: Image Processing in {P}ython. *PeerJ* **2014**, *2*, e453. https://doi.org/10.7717/peerj.453.

(5)	Pelt, D. M.; Gürsoy, D.; Palenstijn, W. J.; Sijbers, J.; De Carlo, F.; Batenburg, K. J. Integration of TomoPy and the ASTRA Toolbox for Advanced Processing and Reconstruction of Tomographic Synchrotron Data. *J. Synchrotron Radiat.* **2016**, *23* (Pt 3), 842–849. https://doi.org/10.1107/S1600577516005658.

(6)	Gürsoy, D.; De Carlo, F.; Xiao, X.; Jacobsen, C. TomoPy: A Framework for the Analysis of Synchrotron Tomographic Data. *J. Synchrotron Radiat.* **2014**, *21* (Pt 5), 1188–1193. https://doi.org/10.1107/S1600577514013939.

(7)	Sofroniew, N.; Lambert, T.; Evans, K.; Nunez-Iglesias, J.; Bokota, G.; Winston, P.; Peña-Castellanos, G.; Yamauchi, K.; Bussonnier, M.; Doncila Pop, D.; Can Solak, A.; Liu, Z.; Wadhwa, P.; Burt, A.; Buckley, G.; Sweet, A.; Migas, L.; Hilsenstein, V.; Gaifas, L.; Bragantini, J.; Rodríguez-Guerra, J.; Muñoz, H.; Freeman, J.; Boone, P.; Lowe, A.; Gohlke, C.; Royer, L.; PIERRÉ, A.; Har-Gil, H.; McGovern, A. Napari: A Multi-Dimensional Image Viewer for Python. Zenodo November 2022. https://doi.org/10.5281/zenodo.7276432.

(8)	Berova, V.; Manjón, A. G.; Paredes, M. V.; Schwarz, T.; Rivas, N. A.; Hengge, K.; Jurzinsky, T.; Scheu, C. Influence of the Shell Thickness on the Degradation of Ru@Pt Core-Shell Catalysts in PEM Fuel Cells. *J. Power Sources* **2023**, *554*. https://doi.org/10.1016/j.jpowsour.2022.232327.
